# Fat Quantification Imaging and Biophysical Modeling for Patient-Specific Forecasting of Microwave Ablation Therapy

**DOI:** 10.3389/fphys.2021.820251

**Published:** 2022-02-03

**Authors:** Frankangel Servin, Jarrod A. Collins, Jon S. Heiselman, Katherine C. Frederick-Dyer, Virginia B. Planz, Sunil K. Geevarghese, Daniel B. Brown, Michael I. Miga

**Affiliations:** ^1^Department of Biomedical Engineering, Vanderbilt University, Nashville, TN, United States; ^2^Vanderbilt Institute for Surgery and Engineering, Vanderbilt University, Nashville, TN, United States; ^3^Department of Radiology and Radiological Sciences, Vanderbilt University Medical Center, Nashville, TN, United States; ^4^Department of Surgery, Vanderbilt University Medical Center, Nashville, TN, United States; ^5^Department of Neurological Surgery, Vanderbilt University Medical Center, Nashville, TN, United States; ^6^Department of Otolaryngology-Head and Neck Surgery, Vanderbilt University Medical Center, Nashville, TN, United States

**Keywords:** liver, hepatocellular carcinoma, fatty liver disease, microwave ablation, finite element, computational model, dielectric, thermal

## Abstract

Computational tools are beginning to enable patient-specific surgical planning to localize and prescribe thermal dosing for liver cancer ablation therapy. Tissue-specific factors (e.g., tissue perfusion, material properties, disease state, etc.) have been found to affect ablative therapies, but current thermal dosing guidance practices do not account for these differences. Computational modeling of ablation procedures can integrate these sources of patient specificity to guide therapy planning and delivery. This paper establishes an imaging-data-driven framework for patient-specific biophysical modeling to predict ablation extents in livers with varying fat content in the context of microwave ablation (MWA) therapy. Patient anatomic scans were segmented to develop customized three-dimensional computational biophysical models and mDIXON fat-quantification images were acquired and analyzed to establish fat content and determine biophysical properties. Simulated patient-specific microwave ablations of tumor and healthy tissue were performed at four levels of fatty liver disease. Ablation models with greater fat content demonstrated significantly larger treatment volumes compared to livers with less severe disease states. More specifically, the results indicated an eightfold larger difference in necrotic volumes with fatty livers vs. the effects from the presence of more conductive tumor tissue. Additionally, the evolution of necrotic volume formation as a function of the thermal dose was influenced by the presence of a tumor. Fat quantification imaging showed multi-valued spatially heterogeneous distributions of fat deposition, even within their respective disease classifications (e.g., low, mild, moderate, high-fat). Altogether, the results suggest that clinical fatty liver disease levels can affect MWA, and that fat-quantitative imaging data may improve patient specificity for this treatment modality.

## Introduction

While many cancers have decreased in incidence over the last more than two decades, primary liver cancer has increased, tripling in the United States since 1980 and rising on average ∼2% per year for much of this time period (an estimated 42,230 new United States cases in 2021 and worldwide are 20-fold greater) (Siegel et al., 2021). This rise has been primarily attributed to obesity, metabolic syndrome, and diabetes, with non-alcoholic fatty liver disease (NAFLD) rapidly replacing viral- and alcohol-related factors as a leading promoter of hepatocellular carcinoma (HCC, the most common primary liver cancer) (Marengo et al., 2016; Masuzaki et al., 2016; Singh M. K. et al., 2018). NAFLD is characterized by an influx of free fatty acids and accumulation of triglycerides in hepatocytes resulting in a lipotoxic environment (Kaufmann et al., 2021). This lipotoxic environment promotes hepatocytes to release reactive oxygen species (ROS) and fibrogenic mediators, which induce hepatic satellite stem cells and stimulate fibrogenic expressions of myofibroblasts (Zhou et al., 2015). This process is known as fibrogenesis and ultimately results in liver cirrhosis. While HCC and cirrhosis are common, in recent reports, a significant portion of HCC now develop without cirrhosis but with FLD (Zoller and Tilg, 2016; Geh et al., 2021) [e.g., ∼20–40% in Kanwal et al. (2018) and Tobari et al. (2020)]. For these patients, early-stage tumors usually present as solitary lesions, and curative treatments, when possible, can be successful. However, detection at a more advanced disease stage, which is common, leads to advanced disease treatment pathways (Masuzaki et al., 2016; Tobari et al., 2020). The consequence of this evolving disease environment is that the management of HCC continues to be a formidable challenge (Masuzaki et al., 2016; Geh et al., 2021).

Currently, there is considerable interest in microwave ablation (MWA) for the locoregional treatment of HCC. In contrast to the historically used radiofrequency ablation (RFA), the enthusiasm for MWA has stemmed from the improved speed, dose delivery characteristics, and outcomes of this treatment (Izzo et al., 2019). One could certainly anticipate that thermal therapies like MWA could be affected by infiltrative fat or the presence of fibrosis. It is well known that dielectric and thermal properties differ between healthy liver and fat with permittivity, electrical, and thermal conductivity being ∼4x, ∼8x, and ∼2.5x greater for healthy liver over fat, respectively (Hasgall et al., 2018). FLD can also vary spatially in liver tissue, i.e., diffuse, diffuse with focal sparing, or focal with largely normal liver (Hamer et al., 2005, 2006). In recent simulation work by Lopresto et al. (2017), investigators estimated the impact of uncertainty in dielectric and thermal properties (∼±25%) with an ablation simulation platform. In these models, ablation predictions were estimated to vary 27% in length and 7% in diameter. Others have pointed to variability due to tissue properties from disease pathology. For example, in a computational modeling study by Deshazer et al. (2016), they report differences in ablations in the presence of fatty liver and fibrosis with increases in ablation volume of 27 and 36%, respectively, over healthy tissue. In Heerink et al. (2018), the investigators build on this study by looking at ablation outcomes with RFA and MWA in the context of HCC and metastatic lesions (*n* = 90 liver tumors). The authors state that “these data clearly demonstrate that the manufacturers’ algorithms based solely on power level and duration of application need to be adapted to the type of tumor in its specific environment.” Another study by Young et al. (2020), involving 86 patients and 103 instances of MWA, identified that greater than 50% of HCC treated resulted in an ablation that was either <85% or >115% of the prediction provided by the manufacturer. The authors report anterior-posterior and transverse dimensions that significantly differed from manufacturer predictions and that correlated with the presence of NAFLD, and fibrosis, respectively. Interestingly, in Amabile et al. (2017) with similar work and findings to Heerink et al. (2018), these investigators discussed the possible role of *quantitative imaging to discriminate differences*. Although it seems clear that the data support a link between outcomes and parenchyma characteristics, current studies are quite disparate, and a potential link to pre-procedural imaging has not been explored.

Microwave ablation simulation platforms that use numerical methods to solve multiphysics differential equations coupling electromagnetic wave propagation and biological heat transfer have been instrumental in testing and validating ablation hardware (Sebek et al., 2017), investigating patient-specific ablation planning (Collins et al., 2015, 2020; Deshazer et al., 2015), and ascertaining the impact of tissue changes (Deshazer et al., 2016; Lopresto et al., 2017). Other groups such as Yoo (2004) and O’Rourke et al. (2007), have recognized that obtaining dielectric and thermal parameters based on disease state is challenging, but resolving this issue can enable accurate forecasting of ablation margins. While the impact of disease states such as FLD and cirrhosis can be simulated and considered, it is not currently feasible to obtain the exact thermal and electrical property estimates of a patient’s liver. However, in recent years, great strides in magnetic resonance (MR) imaging could potentially remedy this gap. Quantitative MR imaging could introduce a tool to enable *anatomically and materially subject-specific* “*tuning”* of a computational MWA model to accurately forecast intra-procedural thermal dose, such that planning and delivery are enhanced, and disease control is significantly improved.

In previous work, a 2D axisymmetric MWA simulation model, fit to physically measured ablations in a realistic mock-tissue phantom study, was used to establish a material model that linked fat quantitative MR imaging to dielectric and thermal properties (Collins et al., 2020). The material model demonstrated a 93.4 ± 2.2% overlap with true measured ablations zones, and in a leave-one-out prospective validation framework, the material model maintained an overlap fidelity of 86.6 ± 5.2% on average. This paper extends the MWA model to a three-dimensional (3D) patient-specific domain and uses clinical fat quantification exams to establish electrical and thermal properties based on the experience in Collins et al. (2020). Four patients with different clinically measured fatty liver disease levels were acquired (low, mild, moderate, high). The models were generated from segmented MR images, and properties were established by a region-of-interest (ROI) analysis of fat quantification images acquired with a commercial MR pulse sequence. The 3D model with adapted properties was then used in a 915 MHz, 60 W, 15-min ablation simulation. As these clinical exams did not include a tumor at the time of imaging, a 2 cm spherical tumor was virtually added to assess potential changes in ablation extent due to a lesion. For each simulation, material properties were set for the surrounding parenchyma as either no infiltrative fat or among one of the image-derived fatty liver disease levels. Spatially encoded simulated temperature data during the ablation with subsequent Arrhenius integral tissue damage assessment were reported and analyzed among the varying models. Finally, the rate of ablation volume per thermal dose was tabulated and visualized as a function of fatty liver content. To our knowledge, while literature exists on varying parenchymal properties (Prakash, 2010; Faridi et al., 2020; Radjenović et al., 2021; Tucci et al., 2021), this work links clinically related fatty liver disease images to the generation of patient-specific dielectric and thermal properties within the context of an image-to-physical material model and reports the consequent changes in the ablation zone.

## Materials and Methods

This study extends previous work in three key ways: (1) all simulations of microwave ablations were derived from patient-specific anatomies and solved in 3D, (2) each simulation’s dielectric and thermal properties were derived from quantitative MR fat quantification imaging data of the patient with perfusion states being indirectly related based on disease state, and (3) microwave ablation performance sensitivity was evaluated relevant to human clinically-diagnosed disease states that were relevant to the locoregional management of liver cancer. The “Materials and Methods” section is structured to convey an overview of the analysis, the imaging and image processing, a material description of the liver tissue to be used in models presented, and the experimental model analysis to be performed.

### Analysis Overview

**FIGURE 1:**
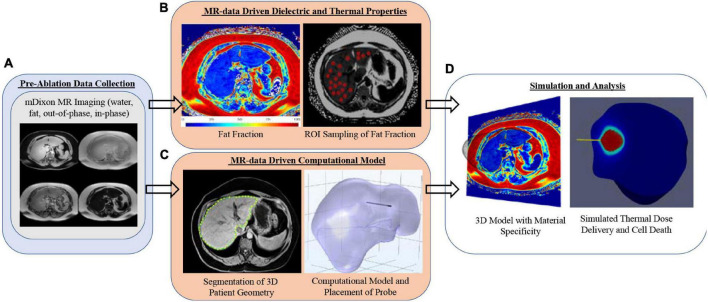
Analysis overview with **(A)** mDIXON MR imaging, **(B)** fat fraction region-of-interest sampling strategy, **(C)** patient-specific computational models with implanted microwave probe, and **(D)** realization of 3D MR-Data-driven patient-geometry-/patient-material- specific computational model with simulated microwave ablation.

**FIGURE 2:**
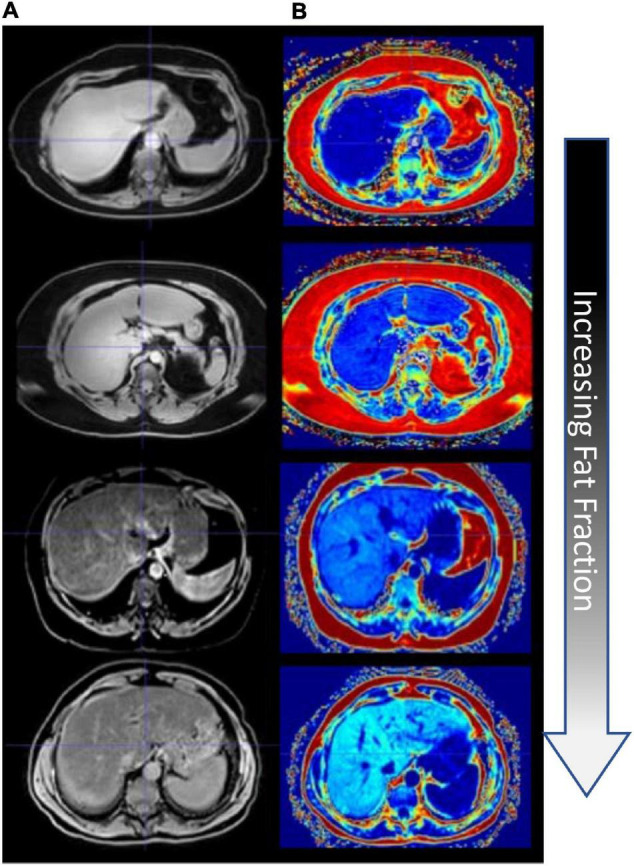
Fat quantification imaging of the liver. **(A)** mDixon water image. **(B)** mDixon fat fraction images (hyperintensity levels indicating increasing fat fraction).

Figure 1 is the framework used in the study reported herein. It begins with the acquisition of patient-specific mDIXON magnetic resonance imaging data (Figure 1A). These data are used within the context of two distinct operations: (1) the determination of spatially localized disease-related biomarkers, i.e., fat quantification, that are proposed surrogates for patient-specific dielectric and thermal properties (Figure 1B), and (2) the generation of a patient-specific three-dimensional computational finite element model of the organ anatomy (Figure 1C). This study hypothesizes that fat quantitative MR images can enable an anatomically and materially subject-specific “tuning” of a computational MWA model to accurately forecast intra-procedural thermal dose (Figure 1D) such that planning and delivery are enhanced, and disease control is significantly improved. If possible, this would be a remarkable advance in the optimization of locoregional thermal ablation therapy delivery. In this work, the framework is employed on four patients with different stages of fatty liver disease (low, mild, moderate, and high). The institutional review board approved this retrospective analysis of de-identified imaging data from the Vanderbilt University School of Medicine.

### Imaging and Image Processing

Patients’ MR images were retrospectively inspected by an experienced radiologist, and subjects with varying levels of fatty liver disease were determined. Imaging data were acquired on one of two clinical scanners — a clinical Philips Intera Achieva 3T MRI or a 1.5T Siemens Magnetom Sola MR scanner. The imaging data collection was part of routine clinical standard-of-care imaging for patients suspected of having fatty liver disease and cirrhosis. A clinically available mDIXON sequence (15.6 ms repetition time, 2.38 ms echo time, 1.1 ms TR) was used to acquire fat, water, in-phase, and out-of-phase image volumes with a 2.083 mm × 2.083 mm × 3.0 mm, and 1.188 mm × 1.188 mm × 3.0 mm voxel resolution over a transverse field of view for the Philips and Siemens scanners, respectively (Eggers et al., 2011; Henninger et al., 2021). Images are shown in Figure 2A, and Figure 2B columns depict the mDIXON water images and fat fraction, respectively, for all four patients.

Liver fat percentage was calculated by sampling the intensity values of liver regions of interest (ROIs) devoid of large blood vessels in the mDIXON transverse slices. ROIs were carefully and manually selected and at least 40 ROIs were sampled per patient liver to calculate an average fat percent. In addition, liver organ geometries were segmented from the mDIXON water scans and post-processed to generate computational organ surface models for all patients. It should also be noted that the patients selected were not liver cancer patients but rather those being surveilled. In order to enhance realism, a location in segment VIII was modified to include a 2 cm diameter tumor that would envelop the tip of the ablation probe. Ablations were compared both with and without tumors, and with and without consideration of material properties derived from quantitative fat imaging data.

### Liver Tissue Material Description

#### Dielectric Properties of Tissue

Dielectric properties of tissue are dependent on the frequency of the probe, in this case, 915 MHz. Electrical conductivity for healthy tissue ranges between 0.79 and 0.88 [S/m] at 915 MHz (Valvano et al., 1985; Kujawska et al., 2014; Hasgall et al., 2018). An electrical conductivity of 0.861 [S/m] was selected as the baseline for tissue with no infiltrative fat from Hasgall et al. (2018). Similarly, at 915 MHz, the electrical conductivity of fat is approximately 0.11 [S/m] from Hasgall et al. (2018). As a result, it would follow that as the liver experiences increased fat infiltration, the overall electrical conductivity would decrease. However, with respect to this process, the constitutive relationship between the functional fat infiltration and organ electrical conductivity is unknown. To address material characteristics, data from previous experiments by Collins et al. (2020) demonstrated a relationship between electrical conductivity and fat within a mock-tissue phantom consisting of agar, albumin, and varying fat levels. This behavior was modified using values from Hasgall et al. (2018) and is presented below in the section “Material Model Construction” (MMC). With respect to HCC tumors, the electrical conductivity has been estimated to be between 0.88–1.26. A study by O’Rourke et al. (2007) estimated that tumor tissue can have an electrical conductivity as high as 26% greater than the organ tissue the cancer resides. Additionally, Stauffer et al. (2003) estimated primary liver tumor electrical conductivity to be 0.88 [S/m] and for metastatic tumors 1.11 [S/m]. For this study, an estimate of 1.26 [S/m] was selected for tumors, corresponding to the 26% increase in electrical conductivity from 0.861 [S/m] of liver tissue with no fat infiltration, as proposed by O’Rourke et al. (2007). With respect to the *relative* permittivity, liver tissue is greater than fat—where estimates range from 45.8–50.8 for liver tissue, and is approximately 10.8 for fat (Stauffer et al., 2003; Hasgall et al., 2018). In this study, the relative permittivity for liver tissue without infiltrative fat was assigned to be 46.8, and similar to electrical conductivity, the relative permittivity for fatty livers was estimated by matching material characteristic behaviors suggested in Collins et al. (2020). The permittivity values from Hasgall et al. (2018) of liver and fat were modified accordingly and are reported in the MMC section below. With respect to HCC tumors, the relative permittivity is on average 26% greater than liver tissue (Stauffer et al., 2003). In Stauffer et al. (2003), primary liver tumors were estimated to be 55.3 and metastatic tumors to be 57.4. In this study, our simulated HCC tissue was assigned a relative permittivity of 55.7, 26% greater than the healthy liver counterpart.

#### Thermal Properties of Tissue

Literature values for thermal conductivity in healthy liver tissue range from 0.48–0.543 [W/(m⋅K)] (Valvano et al., 1985; Guntur et al., 2013; Kujawska et al., 2014; Mohammadi et al., 2021). Papers before 2005 reference thermal conductivity at approximately 0.48 [W/(m⋅K)], but these estimations were derived from tissue experiments at room temperature (25°C) (Valvano et al., 1985). A study from Mohammadi et al. (2021) describes thermal conductivity of *ex vivo* porcine liver as a function of temperature where the thermal conductivity was as high as 0.537 ± 0.009 [W/(m⋅K)] at body temperature (37°C). Another study by Guntur et al. (2013) calculated the thermal conductivity of *ex vivo* porcine liver at 37°C to be approximately 0.520 [W/(m⋅K)]. For the work herein, an estimate of 0.52 [W/(m⋅K)] was selected as the baseline thermal conductivity for tissue with no infiltrative fat, following the values given in Hasgall et al. (2018) and supported by Guntur et al. (2013). With respect to the variations in thermal conductivity as a function of fat content, a similar estimation process that was applied to the dielectric properties was repeated; however, the material model from Collins et al. (2020) reflected a more unique behavior. For simulated tumors, the thermal conductivity average is typically 22% higher than the organ tissue (Ahmed et al., 2008). As a result, the thermal conductivity of HCC tissue was assigned to be 0.624 [W/(m⋅K)].

#### Perfusion Rate

Studies investigating hepatic blood flow estimate hepatic perfusion in healthy liver between 14–18 [kg/m^3^ ⋅s]. Previous studies from Hashimoto et al. (2006) and Zhong et al. (2009) used a triphasic acquisition technique to measure normal hepatic blood flow. Those studies measured an average perfusion of 18 [kg/m^3^ ·s]. Another study by Van Beers et al. (2001) measured CT liver perfusion as low as 9 [kg/m^3^ ·s] in Child C patients and measured variations in cirrhotic liver perfusion, which was approximately 36% lower than values in healthy liver tissue. In this study, 11 [kg/m^3^ ·s] was the lowest possible perfusion value and reflected significant cirrhotic perfusion. In the case of a liver with no infiltrative fat, 18 [kg/m^3^ ·s] was the established perfusion value. While the experiments in Collins et al. (2020) did not reflect a perfused phantom, evidence in the literature demonstrates a correlation between fat fraction and perfusion in the context of fatty liver disease, e.g., Joo et al. (2014) and Troelstra et al. (2021). Unlike the previous dielectric and thermal property strategies where fat fraction directly influences constitutive behavior, perfusion is based on disease state and not material composition *per se*. As a result, while there is an indirect relationship to fat fraction, the establishment of perfusion has a saturation level where no further reduction in perfusion is enabled. In the next section, MMC, the fat-dependent material model construction is discussed, and the values are reported in Table 2.

#### Material Model Construction

Table 1 describes baseline values for liver, fat, and tumor used and taken from Hasgall et al. (2018) with Arrhenius factors taken from Collins et al. (2019). Density (*r*), frequency factor (*A*), and activation energy (*DE*) were maintained in all models. As discussed above, the change in dielectric and thermal properties as a function of fat percentage were estimated from previous mock tissue experiments studying the variation in dielectric properties as a function of fat content within the context of microwave ablation and realistic mock liver tissue phantoms (Collins et al., 2020). More specifically, with respect to dielectric properties, while the results in Collins et al. (2020) were fitted to a linear relationship, the results strongly suggested that a volume fraction weighting of components captured the constitutive model well and was used to create Table 2 values. The only modification from Collins et al. (2020) is that the baseline dielectric properties of liver and fat to create the volume fraction weighting were taken from Hasgall et al. (2018), which reflects human data at 915 MHz. With respect to thermal properties, however, the physical-to-lesion model fitting in Collins et al. (2020) did not reflect this relatively simple behavior but rather a more dramatic effect. Using the baseline values of liver, and fat from Hasgall et al. (2018), and the observed behavior of thermal conductivity from Collins et al. (2020), a material model for thermal conductivity as a function of fat fraction was developed according to,


(1) $$k\left(f\%\right)=\left(\left(k_{liver}-k_{fat}\right)e^{\tau_{k}*f\%}\right)+k_{fat}$$

where *f*%,*k*_*liver*_,*k*_*fat*_ are the fat fraction percentage from imaging data, liver thermal conductivity, and fat thermal conductivity taken from Hasgall et al. (2018), respectively, and reported in Table 2. The value of τ_*k*_ was determined from the phantom experiences of Collins et al. (2020) and in this work was τ_*k*_ = −0.0546. Blood perfusion, ω_*b*_,varied between healthy and cirrhotic liver perfusion values as a function of fat fraction percentage with complete cirrhotic perfusion rates saturating at a fat fraction of 35%. Table 2 lists the dielectric, thermal, and perfusion properties of liver tissue at all disease states. Lastly, while the region of interest (ROI) analysis performed in section “Imaging and Image Processing” expresses a degree of spatial heterogeneity with respect to fat percentage, in this study, the fat fraction across all ROIs at a particular disease level was averaged and then used to set the material properties based on the description above, i.e., Table 2 values were employed over the entire tissue volume.

**TABLE 1:** Material properties of liver, fat, and tumor.

Property	Liver	Fat	Tumor
Heat Capacity at Constant Pressure (*C*_*p*_) [J/(kg⋅K)]	3,400	2,348	3,400
Density (ρ) [kg/m^3^]	1,050	911	1,050
Frequency Factor (A) [1/s]	7.39 × 10^39^	4.43 × 10^16^	7.39 × 10^39^
Activation Energy (ΔE) [J/mol]	2.58 × 10^5^	1.30 × 10^5^	2.58 × 10^5^

**TABLE 2:** Shows the disease state index and the corresponding percent fat derived from the fat quantification imaging data for each patient.

Patient fat content index	0	1	2	3	4		

Disease status	None	Low (0–6%)	Mild (6–17%)	Moderate (17–22%)	High (>22%)	Tumor	Fat
Fat percent (%)	0	3.9 ± 2.3	14.70 ± 3.6	21.20 ± 2.9	29.90 ± 3.7	—	100%
Thermal conductivity [W/(m⋅K)]	0.521	0.461	0.349	0.307	0.271	0.624	0.21
Electrical conductivity [S/m]	0.861	0.831	0.749	0.7	0.634	1.24	0.11
Permittivity	46.8	45.4	41.6	39.3	36.2	55.7	10.8
Perfusion (1/s)	0.018	0.01722	0.01506	0.01376	0.01202	—	—

*Material properties at a particular disease state were determined using the material characteristic curves established in Collins et al. (2020) but with a modified scale based on the human data ranges from Yu et al. (2017) and Hasgall et al. (2018).*

### Experimental Model Analysis

#### Computational Model

A 3D finite-element model was created in COMSOL Multiphysics 5.6 (COMSOL Inc., Burlington, MA, United States) to simulate electromagnetic wave propagation and heat transfer in a patient-specific liver model with a 915 MHz coaxial antenna with a ring slot (Collins et al., 2019). The propagation and absorption of electromagnetic waves radiating from the antenna in the model, assuming no existing charge, is described by Maxwell’s electromagnetic wave equation in three dimensions,


(2) $$\nabla \times \left(\mu_{r}^{-1}\nabla \times \vec{E}\right)-\frac{\omega^{2}}{c_{0}^{2}}\left(\varepsilon_{r}-\frac{j\sigma }{\omega \varepsilon_{0}}\right)\vec{E}=0$$

where the material properties are the relative permeability $\mu_{r}^{-1}$, relative permittivity ε_*r*_, and electrical conductivity σ [S/m]. ω [rad/s] is the angular frequency of the electromagnetic wave, *c*_0_ [m/s] is the speed of light in a vacuum, and $\vec{E}$ [V/m] is the electric field strength. Heat transfer was modeled using Pennes’ Bioheat equation,


(3) $$\rho C_{p}\frac{\partial T}{\partial t}=\nabla \cdot k\nabla T+\rho_{b}C_{p,b}\omega_{b}\left(T-T_{b}\right)+\frac{1}{2}\sigma {\left|\vec{E}\right|}^{2}$$

where ρ [kg/m3] is mass density, *C_p_* [J/kg⋅K] is the isobaric heat capacity of liver tissue, *k* [W/m⋅K] is thermal conductivity, *T* [K] is the current temperature, ρ_*b*_*C*_*p*, *b*_ω_*b*_, and *T*_*b*_ are the density, isobaric heat capacity [J/kg⋅K], perfusion [1/s], and temperature [37°C] of blood, respectively. The last term of equation (3) is heat generation due to absorbed electromagnetic energy in [W/m^3^]. This equation accounts for thermal conductivity, heat storage, and perfusion exchange in a living tissue, modeled as a solid medium. Thermal tissue injury is expressed with the Arrhenius damage integral in COMSOL (1998–2021a). The degree of thermal injury, α, is defined as,


(4) $$\alpha \left(t\right)=\int_{0}^{t^{\prime }}\left(Ae^{-△E/RT\left(t\right)}\right)dt.$$

The degree of tissue injury over time α(*t*), is a function of the frequency factor *A* [1/s], activation energy △*E* [J/mol] to induce tissue damage, universal gas constant *R* [J/mol⋅K], and temperature history of the liver model *T(t)* [K]. The fraction of damaged tissue (θ_*d*_) can then be determined by:


(5) $$\theta_{d}=1-e^{-\alpha \left(t\right)},$$

where θ_*d*_ represents the percentage of cell death, and a threshold of θ_*d*_ > 0.98 was used to indicate cell necrosis. This specific Arrhenius expression has been established to accurately calculate necrosis resulting from hyperthermic damage (Henriques, 1947; Gabriel et al., 1996; Dewey, 2009; Pearce, 2009; Prakash and Diederich, 2012).

#### Boundary Conditions

A first-order electromagnetic scattering boundary condition was applied to the parenchyma of the liver and exterior boundaries of the microwave probe to limit the reflection of the outgoing electromagnetic waves. The equation is,


(6) $$\vec{n}\times \left(\nabla \times \vec{E}\right)-\left(j\kappa +\frac{1}{r}\right)\vec{n}\times \left(\vec{E}\times \vec{n}\right)=0,$$

where $\vec{n}$is the direction normal to the boundary, *r* is the radial axis of the modeling domain (m), and κ is the wavenumber. The antenna is modeled as a conventional conductive core surrounded by a dielectric material and a catheter, with a ring-shaped slot near the tip. The conductive material is expressed with the boundary condition,


(7) $$\vec{n}\times \vec{E}=0.$$

The microwave source is modeled as a port boundary condition adopted from COMSOL (1998–2021b). The microwave probe was modeled after a 915 MHz MicroThermX SynchroWave^®^ ST Antenna (Varian Medical Systems, Austin, TX) and was inserted 65 mm inside the center of Couinaud segment VIII for each patient. A thermal ablation of 60 W continuous power for 15 min was simulated *via* the S-parameter port boundary conditions:


(8) S=∫port 1((Ec-E1)E1*)dA1∫port 1((Ec-E1)E1*)dA1

where *E_c_* is excitation plus the reflected field, *E*_1_is the electric field of the port, and *A*_1_ is the area of the port boundary. Boundaries along the exterior of the phantom were set to the temperature of the human body (37°C). Saline cooling of the antenna was simulated as a convective heat flux condition along the inner boundary of the antenna,


(9) $$\vec{n}\cdot \left(-k\nabla T\right)=h\left(T-T_{ext}\right),$$

where $\vec{n}$is the unit normal, *k* [W/m⋅K] is the thermal conductivity, *h* [W/m^2^⋅K] is the heat transfer coefficient [*h*980 (W/m^2^⋅K) based on past empirical work (Collins et al., 2020)], and *T*_*ext*_ is the saline temperature (20°C).

#### Model Configurations

Using the methods illustrated in Figure 1C, a series of computational model geometries were derived from *n* = 4 patients. To understand performance across patient types, each computational model had a simulated ablation at each of the liver-disease levels (i.e., liver with no infiltrative fat and low, mild, moderate, and high fatty liver disease), resulting in a total of 20 simulations (4 patients × 5 disease states). In addition, these simulations were repeated with the addition of a 2-cm HCC tissue type surrounding the probe for a total of 40 simulations. Properties associated with Tables 1, 2 were utilized in the models.

#### Temperature and Ablation Analysis

To analyze the effects of fat content on ablation temperature, a measurement point was placed 5 mm radially from the ring slot of the probe in each model, similar to previous tissue simulation experiments by Brace (2009). The temperature at this point was recorded over 15 min, and a range representing the extremum was reported with and without a tumor to show the expected temperature range across fat content configurations and dosage. The ablation volume was calculated over time using data exported from COMSOL and processed in MATLAB (MathWorks, Natick, MA, United States). More specifically, as simulations evolved in time, Arrhenius integrals were continuously computed, the spatial coordinates of vertices that established Arrhenius values (θ_*d*_) > 0.98 were extracted, and a mesh volume was created from these coordinates to estimate the necrotic tissue volume. To acquire the temporal course of the necrotic zone development, this process was performed every 15 s over the entire 15-min ablation period. The Arrhenius value threshold was based on similar ablation volume analyses reported in the literature at similar thermal doses and experimental configurations (Deshazer et al., 2016; Collins et al., 2019). The ablation volume (cm^3^) vs. the delivered thermal dose was plotted with varying degrees of fat fraction percentages displayed as an overlay. Although this was a limited patient cohort, ablation volumes were statistically compared amongst the group to detect differences with a series of paired *T*-tests among model ablation configurations. Differences were considered statistically significant at *p* < 0.01, for *a* = 0.05. The final ablation volume, long-axis diameter, and short-axis diameter were then tabulated.

## Results

### Fat Content Analysis

**FIGURE 3:**
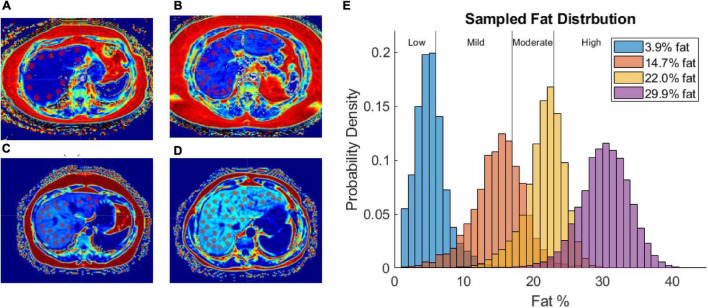
**(A–D)** Fat fraction image segmented with ROIs in patient with low-fat **(A)**, mild-fat **(B)**, moderate-fat **(C)**, and high-fat **(D)**. **(E)** Histogram of fat-encoded intensity values segmented from mDIXON fat fraction images of 4 patients belonging to different liver-fat disease states (Low, Mild, Moderate, High). Average fat percentages are shown in legend.

Fat content was sampled from fat fraction images by capturing 1 cm diameter circular regions of interest (ROIs) devoid of large blood vessels (at least 40 ROIs in all patients). Figures 3A–D shows the distribution of ROIs for each patient in one of the image slices sampled. Figure 3E shows a histogram of the probability density of fat percentages for each patient and the clinically defined thresholds. Of note is the considerable variance in fat percentages within an individual liver for each patient and among the patient cohort.

Table 3 reports the average final ablation volume, long-axis diameter, and short-axis diameter across the models with and without tumors and at each disease state. When comparing the size of necrotic volumes without and with a tumor in the presence of *equivalent disease states*, the volumes were not statistically different in nearly all instances (*p* > 0.01). However, the ablation volume comparisons with respect to the presence of tumor, inside liver parenchyma with no infiltrative fat, was found to significantly differ (*p* = 0.01). When looking at the *effect of fatty liver disease across states*, *most necrotic volumes were tested to significantly differ across disease levels with some exceptions*. The exceptions (i.e., the ablation volumes considered to be statistically identical) occurred when comparing ablation volumes with no-fat to low-fat disease states (*p* = 0.43) and when comparing moderate fat to high-fat disease states (*p = 0.013*)— both in the presence of a tumor. Similarly, there were two exceptions in the scenario associated with ablation without tumor effects: comparing necrotic volumes between parenchyma with no fat infiltration and low-fat disease states, and comparing ablation volumes between low and moderate-fat disease states with *p* = 0.10, and *p* = 0.051, respectively.

**TABLE 3:** Disease status and fat-content range for each patient, with average ± SD of the final ablation volume (cm), long distance diameter (cm), and short distance diameter (cm) with and without a tumor.

Patient Fat Content Index	0	1	2	3	4

Disease status	None	Low (0–6%)	Mild (6–17%)	Moderate (17–22%)	High (>22%)
**Results in models without tumors**					

Long-Axis Diameter (cm)	4.81 ± 0.03	5.01 ± 0.22	5.23 ± 0.22	5.39 ± 0.15	5.53 ± 0.11
Short-Axis Diameter (cm)	2.05 ± 0.05	2.07 ± 0.06	2.19 ± 0.11	2.32 ± 0.11	2.34 ± 0.06
Ablation Volume (cm^3^)	8.50 ± 0.08	8.98 ± 0.49	9.86 ± 0.52	11.19 ± 0.13	11.88 ± 0.31

**Results in models with tumors**					

Long Axis-Diameter (cm)	5.18 ± 0.03	5.19 ± 0.09	5.53 ± 0.07	5.56 ± 0.15	5.67 ± 0.10
Short Axis-Diameter (cm)	2.06 ± 0.06	2.03 ± 0.03	2.04 ± 0.03	2.26 ± 0.08	2.28 ± 0.13
Ablation Volume (cm^3^)	8.96 ± 0.15	9.10 ± 0.31	10.18 ± 0.25	11.57 ± 0.36	12.43 ± 0.33

### Temperature Analysis

**FIGURE 4:**
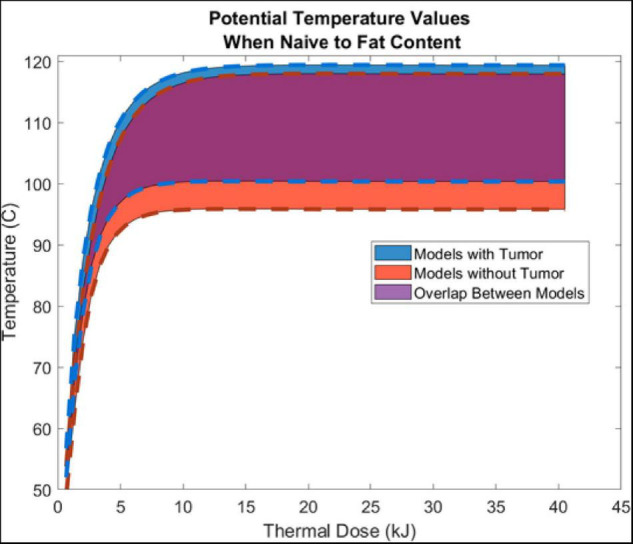
Shows the aggregate average ± SD temperature increase (from all 5 liver-fat configurations) as a function thermal dose (kJ) (Watts⋅s). Temperature data was sampled 5 mm radially from the center of the air slot of the microwave probe (915 MHz probe at 60 W of continuous power for 15 min). Models with tumors are shown in blue, models without a tumor are shown in red, and the overlap between the two are shown in purple.

Thermal history was captured at a point 5 mm radially from the center of the ring slot of the probe. Generally, temperature margins are assumed to be symmetric; and, all models were verified and found to reflect temperature margins that were symmetrically distributed from the center of the probe ring slot. Figure 4 illustrates the temperature evolution at this radial location with increasing thermal dose in models with and without a tumor. The width of the respective temperature envelope reflects the variation in temperature at the respective point due to the different levels of fatty liver disease. Of note is the considerable overlap of the models (purple).

### Ablation Volume Analysis

**FIGURE 5:**
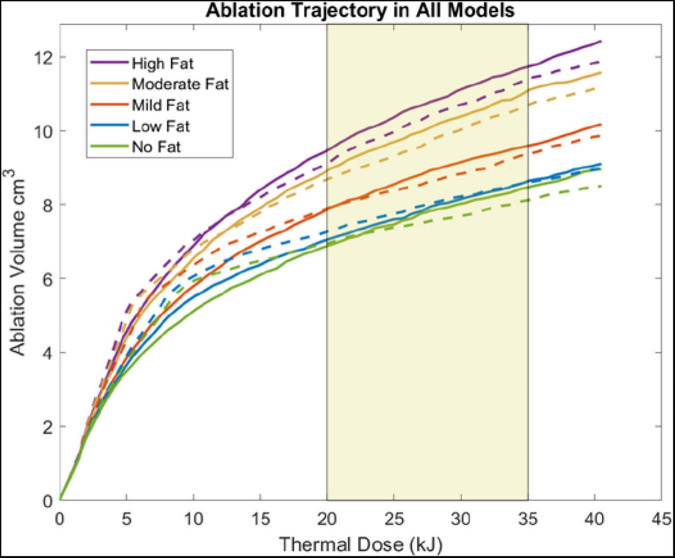
Plots show the ablation volume (cm^3^) as a function of thermal dose (kJ) (Watts⋅s) (915 MHz probe at 60 W of continuous power). Ablation volume captures regions where Arrhenius value ≥ 0.98. Ablation volumes from models without a tumor are shown in dashed lines. Ablation volumes from models with a 2 cm HCC tumor are shown in solid lines. The average clinical thermal dose range is highlighted in *light gold* (Simo et al., 2013; Yu et al., 2017).

**FIGURE 6:**
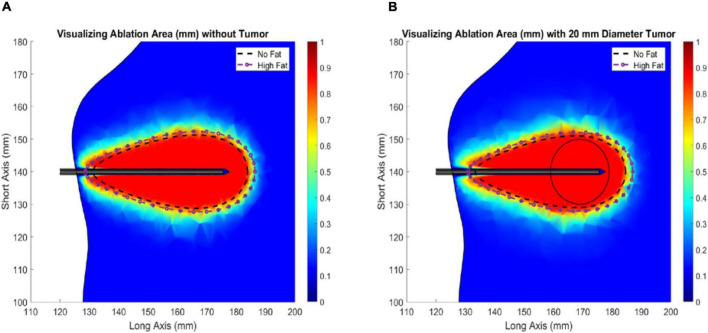
Ablation of liver tissue (915 MHz probe at 60 W of continuous power for 15 min). Ablation margin outlines area where Arrhenius value (*θ_*d*_*) ≥ 0.98. The average ideal liver ablation margins are outlined in *a dashed black line* and the average high-fat (29.9 % liver fat) ablation margins are outlined in *a dashed dotted purple line*. **(A)** Liver models without an HCC tumor. **(B)** Liver models with a 20 mm diameter HCC tumor.

Figure 5 illustrates the change in ablation volume as a function of evolving thermal dose for all five fatty liver configurations without and with tumors averaged over all subjects. Of note is the faster initial trajectory of models without tumors to those with tumors. At later stages in the evolution of thermal dose, ablation volumes with tumors overtake those without tumors, producing larger necrotic volume estimates. Looking across the distribution of disease, considerable growth in necrotic volumes corresponds with an increase in the disease state. To further illustrate the effects of fat content on the ablation zone, the average long-axis and short-axis diameter from no-fat and high-fat were used to fit an ellipsoidal equation to the ablation zone at the end of the 15-min ablation. A direct comparison can be made between ablation zone extents by fitting the ellipsoids in the same manner. Figures 6A,B shows the comparison of these ellipsoids without and with tumor, respectively. The most considerable note is the clear extension of the ablation zone margins in the case of high-level fatty liver disease.

## Discussion

The work reported herein supports observations that tissue thermal and dielectric properties are important parameters to consider in the prediction of microwave ablation margins and provides a rationale to investigate the accuracy and precision of MWA clinical modeling in the context of fatty liver disease. To our knowledge, this is the first simulation study to investigate the use of clinical fat quantification imaging as a source for determining tissue dielectric and thermal properties for the simulation of MWA.

### Ablation and Fatty Liver Disease

In the simulation experiments performed above, four patient-specific liver models were developed, and microwave ablations with a 915 MHz single-slot coaxial microwave probe were simulated. For each patient, a healthy liver parenchyma control with no infiltrative fat was created, accompanied by a series of models with four degrees of fatty liver disease (Table 2). The purpose was to assess the degree of necrotic volume change under different disease states, and more specifically, the size and extent of the ablative zone. The ablation volumes, short-axis, and long-axis diameters were calculated from each set of liver meshes with the same ablation configuration and reported in Table 3. These results provide a few intriguing insights. With respect to the control parenchyma without infiltrative fat (and no presence of tumor), the long-axis and short-axis of the ablation zones can increase by approximately 15.0 and 14.1% at high-fatty liver values, respectively. Additionally, the volume of necrotic tissue can increase by as much as 39.8%. In the presence of the mock tumor, the long-axis and short-axis increased size by 9.5 and 10.7%, respectively, and the necrotic tissue volume increased by 38.7%. This increase in ablation volume with increasing fat has been clinically observed and has often been called the “oven effect” with surrounding low thermally conducting tissue retaining high temperatures near the probe—enhancing ablation. These results are consistent with recent clinical findings demonstrating increased ablative critical diameters in both MWA and RFA in fibrosis and fatty liver disease (Amabile et al., 2017; Heerink et al., 2018; Wang et al., 2020; Young et al., 2020; Tsochatzis et al., 2021) as compared to a liver without these conditions. When comparing the simulations with a tumor to that without a tumor, the ablation volumes with the tumor present increased by approximately 4.6%. Given that the tumor tissue was modeled as more conductive both dielectrically and thermally, this increase is consistent. However, it is interesting that the parenchyma’s material and biological properties affected the ablation volumes eightfold more than the presence of a tumor in the data reported herein.

### Patient Fat Distribution

Figures 3A–D demonstrates the utility of fat quantification MR imaging. The hyperintense areas with increasing fat demonstrate a precise tracking of fatty liver disease. The ROI analysis provided in Figure 3E also quite remarkably demonstrates a significantly distributed representation of fat deposition. While the average liver fat percent was 29.9 ± 3.7% for the patient with high-fatty liver disease, across all ROIs, fat percent levels vary 20–40%. Given the ROI sampling provided in Figure 3D, this variability implies spatial heterogeneity exists in the distribution of fat infiltration for these patients. While in this work, the average fat percent value was used to establish dielectric and thermal properties for the entire organ, the influence of this spatial heterogeneity on thermal evolution requires further investigation. Utilization of image-to-grid methods (e.g., Miga et al., 2000) to realize spatial heterogeneity could be used to study the influence of fat distribution patterns in future work. It should be noted that it is well recognized that fatty liver disease can vary spatially in liver tissue (Hamer et al., 2005, 2006). For example, work by Cheung et al. (2010) identified specific fat patterns, like dorsocervical lipohypertrophy (DCL), to be strongly associated with severity of steatohepatitis, which others have established will change liver material properties and affect ablation extents (Deshazer et al., 2016; Dou et al., 2020). The data in this work suggest a heterogeneous disease-state and that future models investigating ablation should likely incorporate spatially varying material distributions.

### Ablation Temperature Analysis

Results presented in Figure 4 illustrate the range of temperature values at a point 5 mm radially from the ring slot across all patients as a function of thermal dose. As observed in the values of Table 3, Figure 4 demonstrates only modest differences when compared without and with a tumor present. It demonstrates that thermal trajectory is more affected by the change in the parenchyma material properties as a function of fatty liver disease extent than the presence of the tumor. For example, the difference in thermal profiles in the extrema, i.e., comparing the blue and red dashed lines, is considerably smaller than the entire width of thermal differences, caused by the levels of disease present in the simulations study, i.e., the purple overlap region. It should also be noted that comparing these results to similar 915 MHz models in the literature shows similar profiles (Simo et al., 2013; Deshazer et al., 2016; Collins et al., 2020).

### Ablation Volume Analysis

An Arrhenius value (θ_*d*_)greater than 0.98 was assumed to represent complete cell death in the models, and the observed necrotic volumes derived are compatible with previous experiments (Deshazer et al., 2016; Heerink et al., 2018). Deshazer et al. (2016) developed a similar model where the volumes of a healthy and fatty liver were 9.6 and 12.78 cm^3^, respectively, while our models, reported in Table 3, show volumes of 8.98 and 12.43 cm^3^, respectively. The differences in the calculated ablation volumes can be attributed to Deshazer et al. (2016) using temperature-dependent material properties and a temperature threshold to estimate ablation extents rather than the Arrhenius energy equation used here. Additionally, surface effects and thermal boundary conditions could influence the final necrotic volumes. It should be noted, however, that the purpose of the analysis herein is to use simulation to understand the relative impact of disease states as derived from clinical data rather than exact ablation zone size representation.

When analyzing the trajectory of necrotic tissue volumes as a function of thermal dose in Figure 5, the ablation volumes of the models without tumors are initially faster-growing. As dosing ensues, ablation volumes of the simulations with more conductive (electrically and thermally) tumors eventually surpass those without a tumor. This observation is explained in that the “oven effect” ensues early in the simulations with no tumor as the probe is immediately surrounded by fatty tissue. Conversely, in the early stages of simulations with tumors, the more conductive tumor tissue allows energy to be deposited more widely over the larger tumor volume. However, as thermal dosing continues, the more conducting tumor properties enable a deeper penetration of deposited energy, which results in increased necrotic volumes. The intriguing aspect of these results is not the absolute necrotic volumes *per se* but the effect of a different conductive medium surrounding the probe. Coupling that understanding with Figure 6, the clear difference between the spatial ablation extents of a surrounding liver with no infiltrative fat vs. those of one afflicted with fatty liver disease, provides impetus to better understand tissue properties and localization within the context of clinical recurrence, of which work is beginning to emerge (Kaye et al., 2019; Anderson et al., 2021). Lastly, when one considers the altered properties of cancerous tissue in conjunction with the varied spatial arrangements of cancer margins within the context of spatially varying surrounding levels of infiltrative fat, the results suggest that probe placement among tissue types and in spatial reference to tissue-type margins would lead to variability in power deposition and subsequently differences in thermal evolution. The interplay among these factors may contribute significantly to local recurrence in ablative therapies.

### Effect of Perfusion on Ablation Extents

Studies by Schutt and Haemmerich (2008) and Van Beers et al. (2001) report that tissue perfusion is correlated with the disease state, and Van Beers et al. (2001) shows a large range of perfusion for healthy livers and a relatively smaller range for cirrhotic livers. While an exhaustive study of perfusion is outside the scope of this work, it is important to establish some scale of its effect relative to the material property effects established above. To analyze the effects of perfusion on ablation extents, the standard deviations from the perfusion data reported by Van Beers et al. (2001) for normal livers (±0.0057 1/s) and Class C cirrhotic livers (±0.00217 1/s) were applied to the existing perfusion values for the low-fat and high-fat models, respectively (Table 2). The resulting long-axis and short-axis diameters for the ablation margins are reported in Table 4. From this table, in the low-fat liver disease state, ablation extents decreased with increasing perfusion by approximately 3.9 and 3.5 mm in the long-axis and short-axis diameters, respectively, over the span of perfusion values associated with the data by Van Beers et al. (2001). In the high-fat liver disease state, long-axis and short-axis diameters decreased by 1.1 mm and 3.4 mm, respectively. Groups like (Siriwardana et al., 2017) and (Singh S. et al., 2018) have reported that increased perfusion near the ablation zone is correlated with decreased ablation zones, and the results in Table 4 correspond with their findings. Furthermore, when these changes are compared to the long axis and short axis -diameter changes across increasing disease levels in Table 3 (approximately 4.8 mm and 2.5 mm, respectively), *it is easy to recognize that perfusion has a measurable independent effect on ablation extents at a similar scale*. Fortunately, imaging methods to measure perfusion fraction in the context of fatty liver disease are under clinical investigation (Troelstra et al., 2021). It is intriguing to consider the degree that recent imaging advances could be used to influence patient-specific microwave ablation forecasting and delivery.

**TABLE 4:** Disease status (and fat-content range) for each model and the perfusion used, and the average ± SD of the final ablation volume (cm), long distance diameter (cm), and short distance diameter (cm) in models with a tumor.

Patient Fat Content Index	1		4	
Disease status	Low (0–6%)		High (>22%)	
Perfusion (1/s)	0.0116	0.0228	0.01	0.0144
Long-Axis Diameter (cm)	5.54	5.15	5.77	5.66
Short-Axis Diameter (cm)	2.33	1.98	2.43	2.09
Ablation Volume (cm^3^)	12.24	8.70	14.76	10.56

### Limitations

One limitation of the patient selection in this work is that none of the included patients were diagnosed with HCC because fat quantification scans originated from routine surveillance. Additionally, this early work was conducted with many conventional biophysical modeling assumptions and limitations. Regarding the assignment of material properties, the work does not explore all possibilities of property variation. The work relies considerably on a previous study where mock tissue phantoms consisting of agar, albumin, and varying fat were created within the context of microwave ablations and fat quantification imaging (Collins et al., 2020). The most important finding in that work was that dielectric and thermal properties did vary with fat fraction as quantified by imaging. Interestingly, in that work, dielectric properties reflected a simple volume-weighted fraction of constituent component properties, while thermal properties did not. Adapting this behavior to the mock human environment is an assumption underpinning this work and represents a limitation. Further studies with large-scale analyses of simulations, ablation imaging outcomes, and pre-intervention biomarker imaging are needed to better understand material property behaviors in human systems. Another limitation in the work is associated with temperature-dependent material properties, given existing evidence that material properties do seem to evolve as a function of temperature (Guntur et al., 2013). In addition, there have been recent reports regarding the mechanical contraction of surrounding tissue associated with ablation affecting extent determination (Liu and Brace, 2017, 2019). While many of these limitations will need to be overcome to enable clinical use, the work herein certainly supports the rationale for understanding the role of disease-based imaging biomarkers as a means to “tune” the forecasting of patient-specific thermal therapies.

## Conclusion

Albeit limited, ablation is used as a curative treatment for early-stage clinical presentations of HCC (Alabraba et al., 2019). However, and perhaps more noteworthy, non-surgical locoregional treatments, including MWA, have taken on a critical role in disease management to either bridge patients to transplant or to improve quality of life. As in the application presented here, liver cancer has risen dramatically in the past two decades, which has been attributed to metabolic disorders associated with a changing population, namely, the increasing HCC indication of NAFLD (Marengo et al., 2016; Masuzaki et al., 2016; Singh M. K. et al., 2018). This evolving etiological environment, while relatively simple to understand its origin in the population, presents new and formidable challenges in the management of HCC (Masuzaki et al., 2016; Geh et al., 2021). More specifically, because of biophysical property differences induced from these changes in disease presentation, the behavior of hyperthermic therapies becomes altered. This study presents a concept for leveraging image-based biomarkers to improve patient specificity and treatment delivery in relation to fatty liver disease. Additionally, the brightening horizon on relevant liver-based biomarker imaging modalities such as fat quantification, perfusion, elastography, etc., (Troelstra et al., 2021) makes for intriguing possibilities in the patient-specific delivery of hyperthermic therapies. This ability to link quantitative imaging data to therapeutic delivery is critical when considering the adaptation of therapies in the context of an evolving disease. The work herein provides suggestive evidence that quantitative imaging biomarkers associated with patient-specific therapy tuning may serve as an important direction in optimizing patient care, and for adapting to these disease changes.

## Data Availability Statement

The data analyzed in this study is subject to the following licenses/restrictions: As this is patient data, it is protected. Requests to access these datasets should be directed to michael.miga@vanderbilt.

## Ethics Statement

The studies involving human participants were reviewed and approved by Vanderbilt University Medical Center, Institutional Review Board. Written informed consent for participation was not required for this study in accordance with the national legislation and the institutional requirements.

## Author Contributions

FS performed the modeling and analysis. JC who built the original models in 2D, provide consultation to this work. JH provided assistance in model analysis and comparative analysis. KF-D, VP, and SG are working together to secure imaging data for patients afflicted with FLD. VP and DB are both practicing interventional radiologists and were instrumental in terms of the evaluating the MWA results as compared to clinical experience. MM provided the concept of using MR-derived fat quantification data to seed the development of MWA forecasting techniques. MM also assisted model deployment and oversaw analysis of results. All authors involved in editing.

## Conflict of Interest

The authors declare that the research was conducted in the absence of any commercial or financial relationships that could be construed as a potential conflict of interest.

## Publisher’s Note

All claims expressed in this article are solely those of the authors and do not necessarily represent those of their affiliated organizations, or those of the publisher, the editors and the reviewers. Any product that may be evaluated in this article, or claim that may be made by its manufacturer, is not guaranteed or endorsed by the publisher.
